# Effect of high body mass index on knee muscle strength and function after anterior cruciate ligament reconstruction using hamstring tendon autografts

**DOI:** 10.1186/s12891-018-2277-2

**Published:** 2018-10-10

**Authors:** Wei-Hsiu Hsu, Chun-Hao Fan, Pei-An Yu, Chi-Lung Chen, Liang-Tseng Kuo, Robert Wen-Wei Hsu

**Affiliations:** 1 Sports Medicine Center, Chang Gung Memorial Hospital at Chia Yi, Chia Yi, Taiwan; 2 Department of Orthopaedic Surgery, Chang Gung Memorial Hospital at Chia Yi, No 6 West section, Chia Pu Road, Puzih, Chia Yi Hsien 613 Taiwan; 3 School of Medicine, Chang Gung University, Tao-Yuan, Taiwan

**Keywords:** Body composition, BMI, Leg symmetry index

## Abstract

**Background:**

Increased body mass index (BMI) has been associated with poorer function in patients who have undergone anterior cruciate ligament (ACL) reconstruction. However, the effect of high BMI on muscle strength in these patients remained unclear. The current study aimed to compare knee muscle strength and Knee injury and Osteoarthritis Outcome Score (KOOS) in ACL-reconstructed patients with a variety of different BMIs.

**Methods:**

From November 2013 to March 2016, we prospectively enrolled 30 patients who underwent ACL reconstruction (18–60 years of age). Anthropometric parameters, body compositions, isokinetic muscle strength and KOOS were assessed preoperatively, and at post-operative 16th week and 28th week. The patients were stratified into two groups by BMI, i.e. normal BMI (18.5–24.9 kg/m^2^) and high BMI (≥25.0 kg/m^2^).

**Results:**

Twelve patients in the normal BMI group completed the follow-up, while sixteen patients did so in the high BMI group. In comparison of muscle strength between baseline and 28th week follow-up, the normal BMI group had significant increases in overall knee muscle strength, while the high BMI group only had increases in extensors of uninjured knee and flexors of the injured knee. However, there were significant increases in all KOOS subscales for the high BMI group. The high BMI patients reported increased KOOS, which may reflect the contribution of ligament stability in the presence of inadequate muscle strength.

**Conclusions:**

The normal BMI patients had improvement in all knee muscle strength following ACL reconstruction, while high BMI patients only had increases in certain knee muscles. High BMI patients had a decreased quadriceps muscle symmetry index, as compared to their normal BMI counterparts. Increases in quadriceps muscle strength of the uninjured knee and ACL reconstruction were associated with improvements in KOOS in high BMI patients.

## Background

Anterior cruciate ligament (ACL) reconstruction surgery aims to re-establish the stability in the knee joint and allow patients to return to their previous activities after a period of muscle training [1]. Many factors have been suggested as contributing to self-reported function results for ACL reconstruction, including quadriceps index, pain intensity, and flexion motion deficit [2]. The effect of body mass index (BMI) on long-term outcome for ACL reconstruction patients remained unclear [3]. Increased BMI has been associated with poorer functional outcomes following ACL reconstruction [4, 5]. Meanwhile, ACL reconstruction has also been suggested as an effective treatment irrespective of preoperative BMI [6].

Whether the quadriceps recovers well is a major concern for function following ACL injury and reconstruction, as well as a determining factor in the development of knee osteoarthritis [7–9]. The quadriceps of the injured knee weakens profoundly after anterior cruciate ligament injury and subsequent reconstruction [7, 9–11]. This weakness of the quadriceps muscle has been attributed to arthrogenic inhibition, incomplete voluntary activation of the muscle, and altered quadriceps corticomotor excitability [12–14]. It is generally accepted that the aim of postoperative care should be focused on the recovery of muscle strength after ACL reconstruction [15–17]. In fact, quadriceps strength did affect self-reported knee function [2, 18, 19].

Although it has been found that increased BMI is associated with slower muscle recovery as well as lower quadriceps muscle force in patients following total knee arthroplasty [20], whether this slow recovery occurs in ACL patients was previously unclear. Therefore, the purpose of this study was to compare body compositions, knee muscle strength, and Knee Injury and Osteoarthritis Outcome Score (KOOS) after ACL reconstruction in patients with different BMIs.

## Methods

Between November 2013 and March 2016, we prospectively enrolled 30 patients who underwent anterior cruciate ligament reconstruction**s** (aged 18–60 years) using hamstring tendon autografts. The individuals were excluded if they (1) had a history of surgery to either knee, (2) had a previous partial ACL tear, (3) had other ligamentous damage concurrent with ACL injury, (4) had any complications or additional surgery during follow-up. Since return to activity were usually advised 6 months after ACL reconstruction, end-point assessments were performed at 28 weeks after ACL reconstruction [21, 22]. To understand the temporal changes following ACL reconstruction, 16 weeks were further selected. The anthropometric parameters, body compositions, muscle strength and KOOS were assessed preoperatively, and then 16 weeks and 28 weeks post operatively. All participants were Taiwanese from the southern part of the country. The present study was approved by the institutional review board of Chang Gung Foundation (IRB102-0977B), and all study participants provided informed consent to be involved in the study.

### Muscle strength

The muscle strength of the lower extremity including knee flexor/extensor was tested via the HUMAC NORM system (CSMi, U.S.A.), with the mode of concentric/concentric contraction at the angular velocity of 60 degrees/s [11, 15, 23]. The knee flexor/extensor was tested in a seated position. Isokinetic tests were performed 5 times for each examination, and each trial was separated by a rest period of 3 min. In the present study, muscle strength was then normalized for body weight [11]. Leg symmetry index was calculated via the equation ((Injured side maximum force/uninjured side maximum force) × 100%), and compared [12, 24].

### Body composition

Body composition was assessed using an eight-polar tactile-electrode impedance meter (InBody 720; Biospace, Seoul, Korea), which simultaneously recorded body weight, total body fat mass, and lean body mass [25]. BMI (kg/m^2^) was calculated as follows: BMI = weight/height^2^. The participants were divided into two groups stratified by BMI. The classification of high BMI was based on BMI cut-points established by the World Health Organization. Two levels were determined: the normal BMI group (18.5–24.9 kg/m^2^) and the high BMI goup (≥25.0 kg/m^2^) [6, 26].

### Questionnaire

The Knee Injury and Osteoarthritis Outcome Score is a 42-item self-administered knee-specific questionnaire assessing pain (9 items), symptoms (7 items), activities of daily living (ADL, 17 items), function, sports and recreational activities (Sports/Rec, 5 items) and knee-related quality of life (QOL, 4 items) in five separate subscales. All items are scored 0 to 4; for each subscale the scores are converted to a 0 to 100 scale (0 representing extreme knee problems and 100 representing no knee problems) [27].

The Marx activity rating scales is a patient-reported instrument which is determined by measuring some components of physical function that are common to the most sporting activities [28].

### Exercise protocol

It was suggested the importance of improving lower limb muscle strength, starting at an date (2–6 weeks) early after surgery [29–31]. The participants were prescribed home based rehabilitation protocol that started at 4 weeks after surgery. Telephone to track the implementation of this rehabilitation protocol was performed weekly. The rehabilitation program was 1 h stretching and resistance training, with the use of a resistive band instead of weights, 3–5 times per week for 24 weeks. The stretching consisted of eight movements (lower back stretch, upper back stretch, hip stretch, quadriceps stretch, abdomen stretch, lower leg stretch, arm stretch and groin stretch), each held for 20–30 s. The resistance exercises were completed by side bending, biceps curl, leg extension, seated dip, shoulder press, hip abductor/adductor, chest press and leg press. To minimize fatigue, the exercises were alternated between the upper and lower body. Each movement was 4 sets of 15 repetitions. There was a rest interval between sets of 60 s, but no pauses between repetitions.

### Statistical analysis

All data analyses were performed using the Statistical Package for the Social Sciences for Windows (v 20.0; SPSS Inc., Chicago, IL, USA). All continuous data is presented as the mean ± standard deviation (SD). A two-way analysis of variance (ANOVA; group × time) with repeated measures was used to determine the effects of BMI group and time on the dependent measures. The level of significance was set at *p* ≤ 0.05. A power of 95% and relevant effect size of 0.31 was calculated.

## Results

Twenty-eight patients completed the follow-up test at 28 weeks, including twelve patients in the normal BMI group (BMI = 18.5–24.9 kg/m^2^) and sixteen in the high BMI group (BMI ≥25.0 kg/m^2^) (i.e., at least 75% of the training sessions). The reasons for loss of follow-up in the present study were personal factors, such as moving house. The demographic data showed no differences between both groups other than body weight (Table 1). However, it was found that high BMI patients underwent surgical interventions in a shorter interval after injury, and suffered an increased rate of concomitant meniscal injury. Causes of initial injury include: sports (basketball, volleyball and badminton), falling or car accident.

**Table 1:** Subject characteristics

	Normal BMI group (*N* = 12)	High BMI group (*N* = 16)	*p* value
	Mean±SD	Mean±SD	
Age (years)	41±13	35±13	0.270
Height (cm)	164±9	171±8	0.092
Time from injury to baseline test (mon)	17±28	5±5	0.319
Marx activity rating scales (score)	7±6	7±6	0.700
Male:Female ratio	7: 5	13: 3	0.085
Meniscal damage Yes:No ratio	7: 5	11: 5	0.143
Cause of injury Sport:Other ratio	8: 4	10: 6	0.558

In body composition analysis, we observed a temporary increase in body fat in both the normal and high BMI groups at 16 weeks following ACL reconstruction, then returning to previous level at 28 weeks. Interestingly, an increase in lean body mass was shown in the high BMI group at 28 weeks after ACL reconstruction, which resulted in a corresponding increase in body weight. On the other hand, no difference was shown in the normal BMI group in either lean body weight or fat mass (Table 2).

**Table 2:** Body composition

		Baseline	16 weeks	28 weeks	^‡^*p* value	^§^*p* value	^δ^*p* value
		Mean±SD	Mean±SD	Mean±SD			
Weight (kg)	Normal BMI group	61±9 †	62±10^‡†^	62±10^†^	0.028	0.175	0.263
	High BMI group	85±12	86±12	86±12	0.134	0.214	0.938
	^†^*p* value	< 0.001	< 0.001	< 0.001			
BMI (kg/m^2^)	Normal BMI group	23±2^†^	23±2^†^	23±2^†^	0.641	0.528	0.326
	High BMI group	29±3	29±4	29±3	0.277	0.414	0.775
	^†^*p* value	< 0.001	< 0.001	< 0.001			
LBM (kg)	Normal BMI group	45±8^†^	45±8^†^	45±8^†^	0.672	0.553	0.326
	High BMI group	60±9	60±10	61±10 ^§δ^	0.682	0.017	0.049
	^†^*p* value	0.002	0.003	0.001			
Body fat (kg)	Normal BMI group	16±3^†^	17±3^‡†^	16±4^†^	0.012	0.735	0.141
	High BMI group	25±8	26±8 ^‡^	25±8 δ	0.034	0.865	0.036
	^†^*p* value	0.004	0.004	0.005			
LBM/ body fat ratio (%)	Normal BMI group	291±55	267±60^‡^	291±71δ	0.002	0.994	0.042
	High BMI group	260±84	252±86^‡^	263±86	0.042	0.678	0.073
	^†^*p* value	0.391	0.624	0.391			

*LBM* Lean body mass

^†^*p* ≤ 0.05 between normal and high BMI group

^§^*p* ≤ .05 between baseline and 28 weeks

^‡^*p* ≤ .05 between baseline and 16 weeks

^δ^*p* ≤ .05 between 16 weeks and 28 weeks

**Fig. 1:**
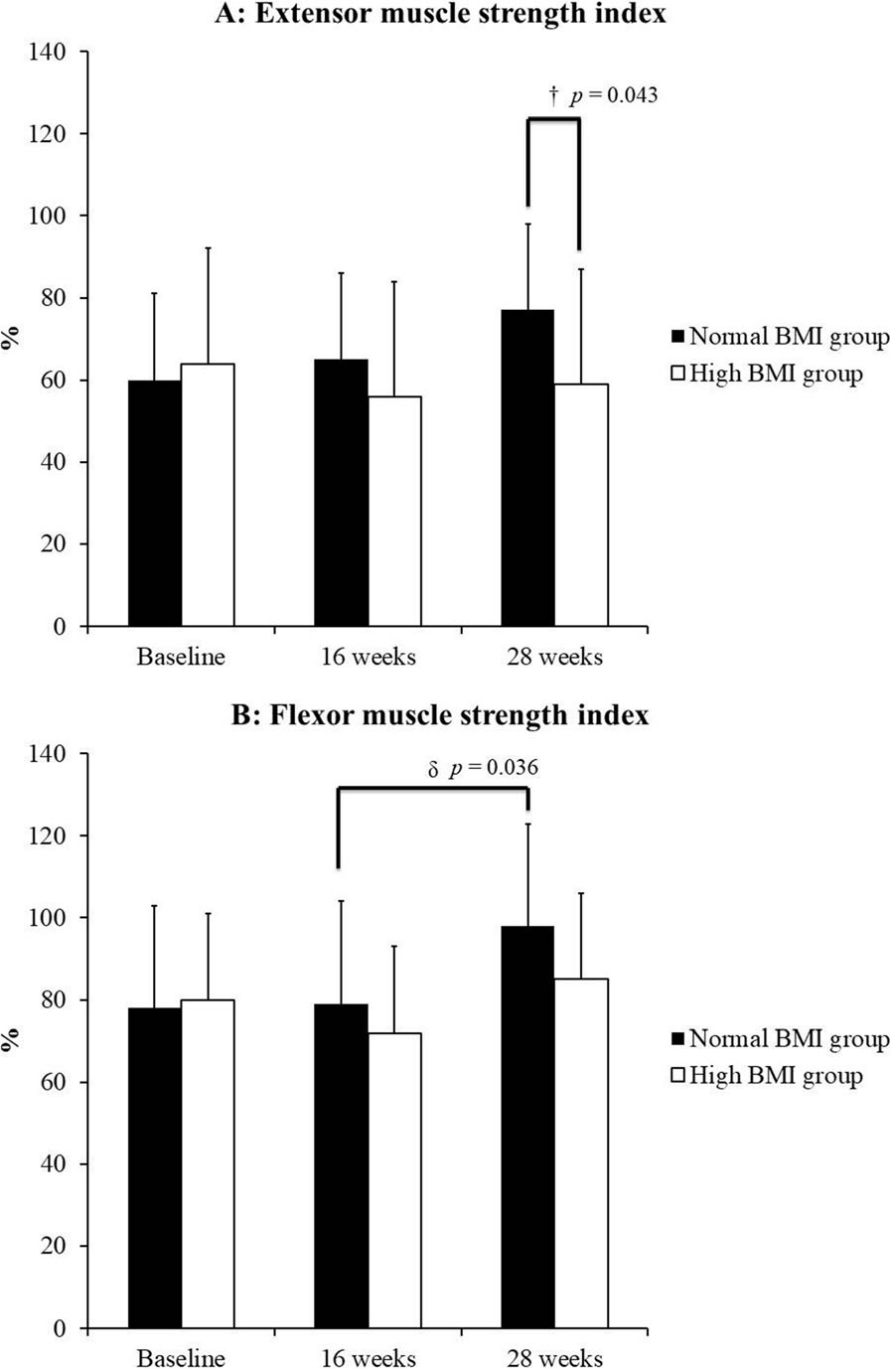
Knee muscle strength index (Injured side/Uninjured side*100%); **a** extensor muscle strength index; **b** flexor muscle strength index. †*p* <  0.05 between normal and high BMI group; δ *p* < .05 between 16 weeks and 28 weeks

In isokinetic muscle strength assessment, knee muscle strength increased for both knee flexor and extensor in the injured knee for the normal BMI group at the 28- week follow-up. At the same time, an increase was shown only in the knee flexor muscle in the high BMI group. For the uninjured knee, the normal BMI group had simultaneously increased strength in both the knee extensor and flexor. On the contrary, an increase was demonstrated in extensor muscle strength for the uninjured knee in the high BMI group, as well as an increase in flexor muscle strength of the injured knee (Tables 3 and 4). When the symmetry index was calculated and compared, the normal BMI group had a significant increase in the symmetry index as compared to the high BMI group at the 28-week follow-up (Fig. 1).

**Table 3:** Modulus knee muscle strength (N-m)

			Baseline	16 weeks	28 weeks	^‡^*p* value	^§^*p* value	^δ^*p* value
			Mean±SD	Mean±SD	Mean±SD			
Injury side	Extensor	Normal BMI group	42±31	65±33^‡^	75±40^§δ^	0.028	0.011	0.042
		High BMI group	64±35	66±36	76±36	0.777	0.213	0.181
		†*p* value	0.152	0.937	0.938			
	Flexor	Normal BMI group	35±18^†^	51±12	66±25 §	0.066	0.011	0.072
		High BMI group	58±26	61±23	73±25	0.671	0.055	0.065
		^†^*p* value	.034	0.287	0.520			
Uninjured side	Extensor	Normal BMI group	76±40	99±30	96±36^§^	0.097	0.004	0.754
		High BMI group	101±39	120±46	131±42^§^	0.068	0.003	0.296
		^†^*p* value	0.152	0.271	0.058			
	Flexor	Normal BMI group	48±16^†^	66±14^‡^	67±20^§^	0.015	0.002	0.921
		High BMI group	75±26	85±29	89±32	0.088	0.094	0.648
		^†^*p* value	0.016	0.109	0.054			

^†^*p* ≤ 0.05 between normal and high BMI group

^§^*p* ≤ .05 between baseline and 28 weeks

^‡^*p* ≤ .05 between baseline and 16 weeks

^δ^*p* ≤ .05 between 16 weeks and 28 weeks

**Table 4:** Relative knee muscle strength (N-m/ kg)

			Baseline	16 weeks	28 weeks	^‡^*p* value	^§^*p* value	^δ^*p* value
			Mean±SD	Mean±SD	Mean±SD			
Injury side	Extensor	Normal BMI group	0.71±0.48	1.05±0.45^‡^	1.23±0.56^§δ^	0.025	0.017	0.050
		High BMI group	0.75±0.37	0.77±0.40	0.88±0.40	0.816	0.224	0.191
		^†^*p* value	0.903	0.105	0.105			
	Flexor	Normal BMI group	0.58±0.30	0.83±0.18^‡^	1.06±0.29 ^§δ^	0.050	0.012	0.012
		High BMI group	0.68±0.27	0.70±0.24	0.84±0.25^§^	0.737	0.019	0.079
		^†^*p* value	0.358	0.209	0.092			
Uninjured side	Extensor	Normal BMI group	1.24±0.53	1.61±0.44	1.58±0.50^§^	0.093	0.017	0.889
		High BMI group	1.18±0.37	1.38±0.45	1.52±0.44^§^	0.098	0.004	0.301
		^†^*p* value	0.759	0.178	0.806			
	Flexor	Normal BMI group	0.80±0.21	1.08±0.24^‡^	1.09±0.25^§^	0.028	0.012	1.000
		High BMI group	0.87±0.25	0.98±0.27	1.02±0.31	0.098	0.070	0.756
		^†^*p* value	0.426	0.462	0.602			

^†^*p* ≤ 0.05 between normal and high BMI group

^§^*p* ≤ .05 between baseline and 28 weeks

^‡^*p* ≤ .05 between baseline and 16 weeks

^δ^*p* ≤ .05 between 16 weeks and 28 weeks

In the functional assessment via KOOS, there were no differences in any category between the normal and high BMI groups, except in QOL score. The high BMI group had a decreased QOL score as compared to those in the normal BMI group at baseline (*p* ≤ 0.05). These differences in QOL became insignificant at 28 weeks after ACL reconstruction (Table 5). For the high BMI group, the KOOS increased from baseline to 28 weeks after ACL reconstruction in all categories. The symptom score increased from 62 ± 17 to 74 ± 15 (*p* ≤ 0.05). Similar increases were found in pain, ADL and QOL score (Table 5). Meanwhile, the sport/recreational activity score increased from 36 ± 20 to 52 ± 22 at 16 weeks after surgery and further to 64 ± 17 at 28 weeks (*p* ≤ 0.05). For the normal BMI group, an increase in the pain subcategory was demonstrated from 75 ± 15 at 16 weeks to 84 ± 13 at the 28-week follow-up (Table 5).

**Table 5:** KOOS

		Baseline	16 weeks	28 weeks	^‡^*p* value	^§^*p* value	^δ^*p* value
		Mean±SD	Mean±SD	Mean±SD			
Symptoms	Normal BMI group	67±16	68±19	71±16	0.889	0.483	0.528
	High BMI group	62±17	65±18	74±15 §δ	0.594	0.023	0.023
	^†^*p* value	0.407	0.519	0.690			
Pain	Normal BMI group	78±17	75±15	84±13 δ	0.735	0.400	0.027
	High BMI group	74±19	80±14	87±10 §δ	0.605	0.001	0.008
	^†^*p* value	0.602	0.481	0.407			
ADL	Normal BMI group	83±19	87±12	91±11	0.401	0.128	0.108
	High BMI group	78±20	81±18	91±8^§δ^	0.326	0.012	0.004
	^†^*p* value	0.443	0.408	0.443			
Sports/Rec	Normal BMI group	50±30	55±17	71±19	0.623	0.089	0.090
	High BMI group	36±20	52±22+	64±19^§^	0.018	0.001	0.097
	† *p* value	0.207	0.517	0.422			
QOL	Normal BMI group	53±16^†^	58±18	65±12	0.344	0.131	0.235
	High BMI group	38±11	52±19^‡^	58±23^§^	0.008	0.002	0.274
	^†^*p* value	0.010	0.338	0.306			

*ADL* Activities of Daily Living, *Sports/Rec* function, sports and recreational activities, *QOL* knee-related Quality of Life

^†^*p* ≤ 0.05 between normal and high BMI group

^§^*p* ≤ .05 between baseline and 28 weeks

^‡^*p* ≤ .05 between baseline and 16 weeks

^δ^*p* ≤ .05 between 16 weeks and 28 weeks

## Discussion

The present study found that high BMI patients (BMI ≥ 25) had improving quadriceps muscle strength in the un-injured knee, while normal BMI patients had improvements in the both injured and uninjured knees. This study parallels the literature, in that normal BMI patients have an extensor symmetry index approaching 80% at 6-month follow-up [15, 32, 33]. However, such improvement was not shown in high BMI patients. Second, preoperative quality of life was shown inferior in the high BMI group as compared to the normal BMI group, and improved at 28 weeks after ACL reconstruction. Similarly, the score improved in symptoms, pain, sports, and ADL in the high BMI patients. Interestingly, the normal BMI group had only improvement in the pain subcategory, despite recovery of better muscle strength.

Utilizing strength/body weight ratio, we observed that both the normal and high BMI groups have similar flexor and extensor strength for the uninjured knee at 28 weeks follow-up. It seemed that the strength/ body weight would reach a plateau either in the high or normal BMI patients in the uninjured knee, while a persistent deficit was observed in the injured knee and to a greater extent in the high BMI group. The injured knee showed a pattern where the normal BMI group had progressive increases in both knee flexors and extensors, reaching symmetry indexes of 77% and 90%, respectively, while the high BMI group had no significant increases in extensor strength. Hence, the symmetry index for the extensor muscle was shown a significant difference between the high and normal BMI groups at the 28 week follow-up.

Literature suggests that the deficit in quadriceps strength for ACL deficient patients is greater than the deficit in hamstring strength [32, 34, 35]. This deficit in strength can be improved by an exercise program after ACL reconstruction, and recovery of strength in the flexor is faster than in the extensor [36, 37]. The present study exhibited similar patterns, and further showed that high BMI patients had a slower recovery of extensor muscle strength. The muscle strength deficit may result from arthrogenic muscle inhibition or quadriceps corticospinal excitability [13, 14]. It has been suggested that the cortical silent period (cSP) duration is longer in the injured limb of the ACL group, as compared to the uninjured limb [13]. The current study has shown that high BMI was associated with a greater duration of muscle strength deficit. Changed activation of quadriceps and with altered neuromuscular control was shown in obese patients [38]. The quadriceps symmetry index did not reach the desired high quadriceps index (> 90%) that which is suggested to be biomechanically advantageous after ACL reconstruction [8, 19, 21]. Neuromuscular training incorporating motor learning principles, such as eccentric training which was most effective in restoring quadriceps strength, should be added to strength training to optimize outcome measurements [39].

Increasing BMI was associated with a progressive reduction in physical functions [40, 41]. The deficit extended to those patients suffered from musculoskeletal disorders such as arthropathy and ACL ruptures. Surgical interventions, i.e. total knee arthroplasty (TKA) and ACL reconstructions, would result in comparable functional improvement irrespective of different BMIs [6, 42–44]. Interestingly, effect of BMI on pain for TKA and ACL reconstructed patients was shown a similar pattern. In TKA patients, preoperative WOMAC (Western Ontario and McMaster Universities Osteoarthritis Index) pain score was worse as the BMI increased. TKA surgery resulted in a faster improvement in the higher BMI group at 6 month but reached a comparable level across all BMIs at 2 year [45]. In ACL reconstructed patients, faster improvement of pain score was also observed in high BMI patients [6]. In the present study, we demonstrated a significant improvement in KOOS pain subscales in 6 month follow up in the high BMI groups. In addition, we further showed that the high BMI group had improvement in all KOOS subscales, along with an increased muscle strength in the flexor of the injured knee. This could be attributed to, as least in part, the fact that the high BMI group had lower preoperative scores. In the high BMI group, ACL reconstruction providing for re-establishment of static stabilization, along with increased flexor muscle strength, may contribute to the improvement of KOOS. Increased knee muscle strength, mainly in the knee flexors, has been associated with lower activity limitations [46].

The current study was limited by the small number of patients and the relatively short follow-up. Longer-term follow-up is warranted to investigate muscle strength and functional recovery after ACL reconstruction for both normal and high BMI groups. However, this study demonstrated decreased quadriceps muscle strength recovery in the ACL reconstructed knee for higher BMI patients than for normal BMI patients at 6 months follow-up. It is suggested that further rehabilitation should include resistance training and neuromuscular facilitation in high BMI ACL reconstruction patients.

## Conclusion

In the current study, the normal BMI patients had improvement in all knee muscle strength following ACL reconstruction, while high BMI patients only had increases in certain knee muscles. High BMI patients had a decreased quadriceps muscle symmetry index, as compared to their normal BMI counterparts. Increases in quadriceps muscle strength of the uninjured knee and ACL reconstruction were associated with improvements in KOOS in high BMI patients.

## Abbreviations

ACL: Anterior cruciate ligament
ADL: Activities of daily living
BMI: Body mass index
cSP: Cortical silent period
KOOS: Knee injury and Osteoarthritis Outcome Score
QOL: Knee-related quality of life
Sports/Rec: Function, sports and recreational activities
TKA: Total knee arthroplasty
WOMAC: Western Ontario and McMaster Universities Osteoarthritis Index
